# The Human Virome in Infectious Diseases: Insights from Chronic and Acute Infections Across Body Sites—A Narrative Review

**DOI:** 10.3390/microorganisms14050969

**Published:** 2026-04-25

**Authors:** Rebecca Feletti, Antonio Mori, Amina Zaffagnini, Concetta Castilletti, Elena Pomari

**Affiliations:** 1 National PhD Programme in One Health Approaches to Infectious Diseases and Life Science Research, Department of Public Health, Experimental and Forensic Medicine, University of Pavia, 27100 Pavia, Italy; rebecca.feletti@sacrocuore.it; 2 Department of Infectious, Tropical Diseases and Microbiology, IRCCS Sacro Cuore Don Calabria Hospital, 37024 Negrar di Valpolicella, Italy; antonio.mori@sacrocuore.it (A.M.); amina.zaffagnini@sacrocuore.it (A.Z.); concetta.castilletti@sacrocuore.it (C.C.)

**Keywords:** human, virome, viruses, metagenomics, infections, diagnostics, epidemiology

## Abstract

The human virome, comprising eukaryotic viruses, bacteriophages, and viral genetic material, is a dynamic component of the microbiome with growing relevance in infectious diseases. This narrative review is structured to: (i) summarize the general composition of the human virome and methodological challenges, including the fraction of unclassified viral “dark matter”; (ii) describe virome alterations in chronic infections; and (iii) explore site-specific virome dynamics across respiratory, intestinal, and genito-urinary tracts in both chronic and acute infections. In chronic viral infections such as HIV, HBV, HCV, and HPV, a recurrent feature is the expansion of *Anelloviridae*—particularly torque teno virus—reflecting impaired immune surveillance rather than direct pathogenicity, suggesting their potential as surrogate biomarkers of immune competence. Evidence on virome changes in chronic bacterial and parasitic infections remains limited, highlighting a critical knowledge gap. Acute infections are associated with compartment-specific shifts in eukaryotic viruses and bacteriophage communities, often paralleling changes in bacterial populations and inflammatory responses, with implications for disease severity. Despite advances in metagenomic approaches, a substantial proportion of viral sequences remains unclassified, limiting functional interpretation. Nevertheless, virome profiling provides an ecosystem-level perspective, offering insights beyond single-pathogen detection and supporting emerging applications in diagnostics, immune monitoring, prognosis, and infectious disease surveillance.

## 1. Literature Review Methodology

Literature searches were conducted in PubMed to identify articles published over the past 15 years, up to February 2026. The following search terms were used: “virome”, “microbiome”, “metagenomics”, “next generation sequencing”, “infectious diseases”, “respiratory infection”, “intestinal infection”, “chronic infection”, “sepsis”, and “parasitic infection”.

All retrieved articles were screened to ensure that they focused on metagenomic approaches, with particular focus on virome, and that full-text versions were available in English.

Once identified, relevant studies were further assessed for eligibility, and their bibliography were manually reviewed to identify additional studies.

## 2. Introduction

**Figure 1:**
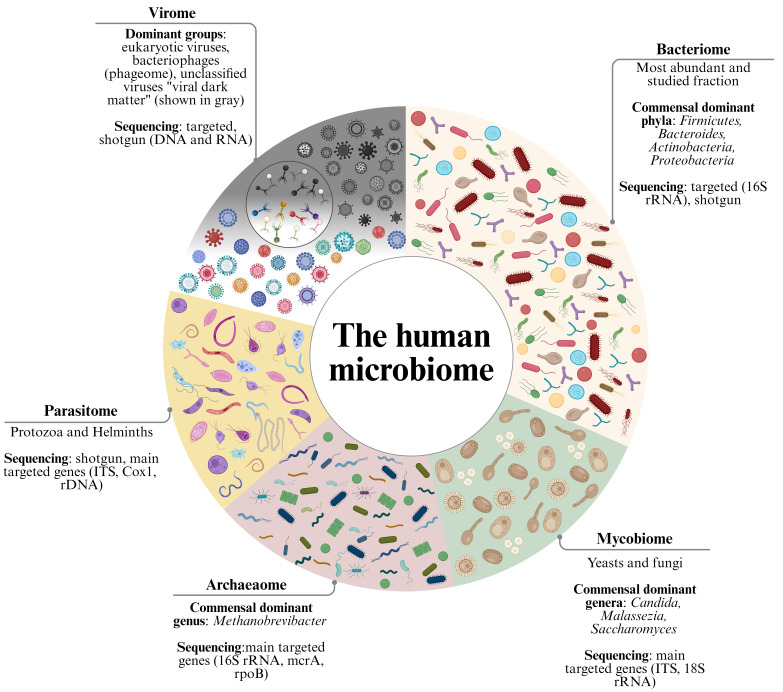
**Structure of the human microbiome.** The human microbiome is composed of multiple distinct subdomains: the ‘**bacteriome’**, representing the dominant bacterial fraction; the ‘**mycobiome’**, encompassing the genomic content of fungi; the ‘**parasitome’**, representing parasites; the ‘**archaeome’**, including the genomic content of archaea; and the ‘**virome’**, which comprises viral genomes. Within the virome, the ‘phageome’ consists of bacteriophages that specifically infect resident bacteria. This subdivision highlights the complexity of microbial communities. Each subdomain is represented along with some representative taxa and the main sequencing approaches used for their characterization, including targeted markers (genomic DNA, nuclear and mitochondrial) and shotgun metagenomics of DNA and RNA. Created in BioRender. Rebecca Feletti. (2026) https://BioRender.com/.

The human body constitutes a highly dynamic ecosystem inhabited by a diverse consortium of microorganisms. This diverse consortium includes bacteria, archaea, fungi, parasites and viruses that play critical roles in health and disease [1,2,3,4]. This consortium is not a simple collection of organisms but a complex and interactive system that influences everything from our metabolism to our immune system [5,6,7]. The microbiome refers to the collective genetic material of these microorganisms and represents a complex, interactive system rather than a simple assemblage of species. Within the microbiome, the virome represents a distinct and functionally unique subcomponent (Figure 1 and Table S1), rather than merely a collection of pathogenic viruses.

Studying the virome is crucial, especially in the context of disease. Beyond their effects on bacterial populations, viruses may also modulate host immune responses through direct interactions with epithelial and immune cells [8]. Bacteriophages and their components can interact directly with host cells, potentially modulating immune responses and contributing to homeostasis of different districts (e.g., intestinal) [9,10,11]. Experimental evidence indicates that bacteriophages can access host tissues via enteric epithelial or immune cell uptake [12], where their components may activate innate immune pathways, including Toll-like receptors (e.g., TLR9), cytosolic DNA sensing via the cGAS-STING pathway, and NOD-like or RIG-I-like receptors leading to downstream activation of inflammasomes (e.g., NLRP3, AIM2) and the NF-kB pathway, thereby modulating mucosal and systemic immune responses [10,13]. However, most of the current evidence derives from in vitro systems and animal models, with comparatively limited and largely associative data in humans [9,11,12,14]. In the literature, the majority of virome studies are observational; however, thanks to the increase in high-throughput sequencing technologies and advanced bioinformatic tools, virome characterization is shifting from exploratory research toward clinical application, particularly in the context of recurrent or chronic infections, immunocompromised patients, and cancer prevention [15,16,17,18,19]. Understanding virome dynamics in different infectious contexts may improve diagnosis, treatment selection and disease monitoring, especially where standard virological diagnostics fail to explain clinical symptoms [20,21].

The overall aim of this narrative review is to summarize and describe reported patterns of virome composition across infectious conditions. The review is structured to describe the virome in the context of chronic and acute infections and within different body districts (intestinal, respiratory, genito-urinary), providing a comprehensive overview of this nascent aspect of human healthcare.

## 3. General Composition of the Human Virome

The human microbiome is a complex and heterogeneous ecosystem composed of multiple subdomains, including the bacteriome, archaeaome, mycobiome, parasitome and virome (including phageome) (Figure 1). Each of these components contributes to host physiology and reflects a distinct taxonomic and functional diversity.

From a methodological perspective, this complexity is mirrored by the diversity of sequencing approaches used to characterize the different microbiome components. Briefly, the bacteriome is commonly profiled through 16S rRNA gene sequencing [22,23]. Similarly, the archaeaome is often investigated using 16S rRNA and additionally functional genes such as mcrA [24,25]. The mycobiome is characterized using internal transcribed spacer (ITS) regions and 18S rRNA markers [26,27], and the parasitome relies on markers including ITS, cox1, and 18S rDNA [28,29]. In contrast, the virome is typically explored using both targeted and untargeted approaches, including shotgun metagenomics of DNA and RNA, to capture the full spectrum of viral diversity [30,31].

Within this framework, the virome encompasses the totality of viral nucleic acids, including single- and double-stranded DNA and RNA genomes [32]. The composition of the virome is highly individualized and influenced by host genetics, age, diet, lifestyle, and health status, thereby constituting a dynamic and personalized biological signature [33,34,35]. The human virome is not composed exclusively of pathogenic viruses: a substantial fraction consists of commensal or persistent viruses that establish long-term, often asymptomatic associations with the host [35].

Viruses colonize diverse anatomical sites, including the gastrointestinal tract, skin and oral cavity, and have been detected in a wide range of biological specimens such as blood, cerebrospinal fluid (CSF), respiratory specimens (i.e., saliva, nasopharyngeal swab, broncho-alveolar lavage fluid), feces, urine and vaginal and cervical secretions [35,36].

Our body harbors an enormous number of viruses (around 10^13^ viral particles) most of which are located in the gut [37]. The human virome can be broadly classified into three major categories: eukaryotic viruses (which infect human cells), prokaryotic viruses commonly known as bacteriophages or phages (which infect bacteria) [38,39] and archaeal viruses (which infect archaea) [35,40]. Studies on viruses infecting archaea are still relatively limited; however, these viruses display remarkable structural and genetic diversity, reflecting adaptation to archaeal hosts that often thrive in extreme environments [3,4]. Eukaryotic viruses include both persistent viruses—such as human immunodeficiency virus (HIV), hepatitis B virus (HBV), herpesviruses, and papillomaviruses—which can establish latent infections and remain in the host for life, and transient viruses, such as noroviruses and enteroviruses, which typically cause acute infections. Bacteriophages represent the largest fraction of the human virome [41,42] and are particularly abundant in the gastrointestinal tract, where they play a crucial role in maintaining microbial homeostasis by modulating bacterial populations through lytic and lysogenic pathways [11,43,44,45].

## 4. Virome, Its ‘Dark Matter’ and Methodological Challenges

A defining feature of virome studies is that a substantial proportion of detected viral sequences cannot be taxonomically classified. This fraction, commonly referred to as viral “dark matter”, reflects the limits of current knowledge [46].

Unlike bacteria, parasites or fungi, viruses lack universal genetic markers and may persist in latent or low-replicative states, making their detection and characterization particularly challenging [40]. This knowledge gap is largely attributable to the extraordinary variability of viral genomes and to historical technical limitations in viral detection and annotation.

Traditional molecular diagnostics rely on targeted amplification of known pathogens and therefore underestimate viral communities. In contrast, virome characterization mainly relies on shotgun metagenomic sequencing, often preceded by viral particle enrichment, filtration, nuclease digestion of host nucleic acids, and random amplification [35,47]. Subsequent bioinformatic analysis combines reference-based alignment, de novo assembly, protein homology searches and, more recently, machine-learning classifiers to infer viral origin [48,49]. Protein homology searches and machine-learning approaches are increasingly used not only for detection but also to infer putative functions of unclassified viral sequences, by identifying conserved domains and predictive sequence patterns linked to viral activity [50,51,52].

Collectively, these advances are increasingly helping to clarify not only the composition but also the function of the virome as a dynamic indicator of the physiological state of the host in both health and disease, and recent studies are showing how infections can be associated with changes in viral composition [47,52].

Additionally, it is important to acknowledge key methodological limitations, including the risk of laboratory and environmental contamination [53,54], amplification biases introduced during sample processing [55,56], and strong dependence on incomplete or uneven reference databases [57,58], all of which can affect viral detection, classification accuracy, and downstream interpretation.

## 5. The Virome and Immunology: Anelloviridae as Biomarkers of Immune Competence

The human virome is increasingly recognized as an integral component of host–immune system interactions, extending beyond its traditional description as a collection of pathogenic and commensal viruses. Among its constituents, members of the *Anelloviridae* family, particularly torque teno virus (TTV), represent the most consistently detected viruses across anatomical sites and clinical conditions [59].

Anelloviruses are highly prevalent, non-enveloped, single-stranded DNA viruses that establish persistent infections in humans without evidence of direct pathogenicity [60]. Their replication appears to be regulated by host immune surveillance, particularly cell-mediated immunity. Accordingly, multiple studies have reported higher anellovirus loads in conditions associated with impaired immune function, including chronic viral infections, transplantation, and immunodeficiency states [61,62,63].

In this context, anelloviruses have been proposed as surrogate biomarkers of immune function, reflecting host immune control over persistent viral replication. However, their clinical utility is limited by several factors. First, anellovirus loads lack disease specificity, as increased levels are observed across heterogeneous conditions associated with immune modulation [64,65]. Second, substantial inter-individual variability and compartment-dependent dynamics have been reported, influenced by host factors such as age, genetic background, and environmental exposures [66]. Third, methodological heterogeneity in sampling, sequencing, and bioinformatic workflows limits comparability across studies [64,65,67]. Finally, current evidence remains largely associative, with limited mechanistic understanding of virus–host immune interactions.

Given their recurrent detection across multiple infectious contexts, anelloviruses—particularly TTV—are frequently referenced throughout this review and should therefore be interpreted in light of the methodological and biological considerations outlined above.

## 6. The Virome in Chronic Infections

Virome analysis has emerged as a powerful tool for investigating the complex interplay between viruses and chronic diseases, providing insights that go beyond the identification of single pathogenic agents [68,69,70,71,72,73]. Chronic infections—whether viral, bacterial, or parasitic—represent a major clinical challenge due to their long-term impact on host physiology, immune function, and microbiome homeostasis [74,75]. Among viral infections, HBV, hepatitis C virus (HCV), HIV, Epstein–Barr virus (EBV), cytomegalovirus (CMV), and human papillomavirus (HPV) are among the most clinically impactful, being associated not only with persistent inflammation and immune exhaustion but also with oncogenic transformation and metabolic dysregulation [76,77,78,79,80,81].

Similarly, recent studies showed that chronic bacterial infections caused by *Helicobacter pylori* and *Mycobacteria* can drive sustained inflammatory responses, alter mucosal immunity and promote microbial alterations in the gut and respiratory tract [82,83,84], highlighting the importance of integrating microbiome including virome analyses in clinical studies. Notably, despite the growing interest in virome research, studies specifically investigating the human host virome in the context of chronic parasitic infections remain limited. For pathogens such as *Plasmodium* spp. and *Leishmania* spp., studies on the virome are essentially limited to the viral infection of parasites themselves and vectors, rather than the human host, leaving a knowledge gap regarding potential viral contributions to human disease [85,86]. Overall, understanding the virome in these chronic contexts could reveal previously unrecognized mechanisms of disease pathogenesis and identify novel therapeutic targets.

### Anellovirus Abundance as a Hallmark of Chronic Viral Infections

Increasing evidence indicates that chronic viral infections are associated with profound and persistent remodeling of the human virome across multiple body sites, with a particularly consistent modulation of anelloviruses, reflecting alterations in host immune homeostasis. These changes appear to represent a common biological response to long-term viral persistence rather than infection-specific phenomena. Such alterations are not confined to a single infection but recur across different chronic conditions such as HIV, HBV, HCV and HPV, which collectively represent major global health burdens associated with long-term viral persistence and immune modulation [87,88,89,90,91,92,93,94,95,96,97,98].

Across these infections, a number of shared features have emerged. The most consistent finding is the frequent detection and dynamic modulation of anelloviruses, particularly TTV [47,59,99,100]. These viruses may serve as context-dependent biomarkers of impaired immune control, although their clinical interpretation remains influenced by host status, sample type, and study design [61,62,63,101]. This pattern is evident across diverse biological compartments—from plasma and broncho-alveolar lavage to stool, cervical, and semen samples—where anelloviruses often dominate the viral community when immune surveillance is compromised.

In the context of systemic infections such as HIV, metagenomic analyses of plasma and respiratory mucosal sites reveal complex viral communities comprising not only the primary pathogen but also other viruses such as hepatitis G virus, endogenous retroviruses (HERV), herpesviruses, adenoviruses, and anelloviruses [18,47,102,103,104,105,106,107,108]. This increased virome complexity parallels progressive immune dysfunction and is frequently accompanied by shifts in bacterial microbiota composition, particularly in the gut, indicating a profound reshaping of host-associated microbial networks [109]. A similar phenomenon is observed in chronic liver diseases, where HBV- or HCV-infected patients exhibit co-detection of *Anelloviridae* [110,111]. Although *Hepadnaviridae* or *Flaviviridae* dominate by read count, *Anelloviridae* are consistently the most frequently detected family, underscoring their remarkable persistence and sensitivity to host immunological tone rather than tissue tropism or disease etiology.

Interestingly, even in infections driven by tissue-specific viruses such as HPV, the virome reflects comparable community dynamics. Samples from individuals with chronic HPV infection harbor a variety of co-infecting viruses, including EBV, BK polyomavirus (BKPyV), molluscum contagiosum virus (MCV), and TTV [112]. The relative abundance of these viruses appears to fluctuate in response to HPV dominance, suggesting competitive, permissive, or immune-mediated interactions among viral species sharing the same anatomical niche, rather than simple passive co-presence.

Collectively, these findings support the view that chronic viral infections are associated with ecosystem-level perturbations of the human virome. Rather than isolated infections, they can entail complex and dynamic interactions among multiple viral, bacterial, and host components. The recurrent expansion of anelloviruses across disparate diseases and body sites points to a shared immunological signature of chronic infection—one characterized by reduced immune surveillance and the consequent reduction in control over commensal or latent viruses.

Importantly, this responsiveness to changes in host immunity also underlies the emerging clinical relevance of anelloviruses as potential biomarkers of immune function and therapeutic response. In several settings, including HIV infection, chronic hepatitis, and especially transplantation medicine, TTV abundance has been shown to correlate with immune reconstitution, disease progression, and response to antiviral or immunomodulatory therapies [7,20,113,114,115]. Thus, anellovirus dynamics may provide integrative information on host immune competence that complements conventional virological and immunological markers. Understanding these common virome alterations opens avenues for the use of metagenomic profiling as a diagnostic and prognostic tool, capable of revealing not only the primary etiologic agent but also the broader viral context that shapes disease progression, therapeutic responsiveness, and long-term host outcomes.

## 7. The Virome in Distinct Body Sites

Methodologically, the characterization of site-specific viromes relies primarily on metagenomic sequencing (shotgun or targeted approaches) of nucleic acids extracted from a wide range of biological specimens, including blood, saliva, nasopharyngeal swabs, broncho-alveolar lavage fluid, feces, urine, vaginal and cervical secretions, and skin swabs [35,36]. To date, the virome of the CSF remains poorly characterized, in part because CSF has traditionally been considered a sterile or near-sterile compartment [116]. As a result, most metagenomic studies have focused on pathogen detection in neurological infections [117,118,119,120], whereas systematic and longitudinal characterization of the CSF virome has received limited attention.

Analyses of the hematic virome, as we reported in the previous sections, frequently reveal a dominance of anelloviruses, reflecting systemic immune status [63,99], whereas mucosal and epithelial sites such as the respiratory, gastrointestinal, and genital tracts display highly variable virome structures shaped by local microbial communities, host barriers, and environmental exposures [35,39,121,122,123,124].

These differences highlight that the human virome is not a uniform entity but a network of semi-independent niches interconnected through host physiology and immune regulation [38,125].

From a clinical perspective, the study of compartmentalized viromes holds substantial promise. Understanding how viral communities vary across body sites may identify site-specific biomarkers, guide sampling strategies for diagnostic viromics, and help elucidate how localized viral communities contribute to systemic inflammation, chronic disease progression, or therapeutic response [17,19,71,126,127].

**Figure 2:**
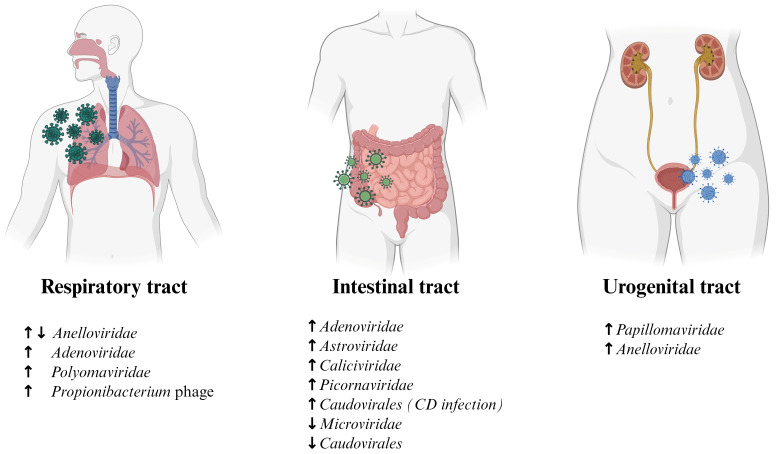
**Observed virome patterns across infections.** Respiratory tract infections often show dynamic fluctuations of *Anelloviridae* abundance (with levels decreasing ↓ during acute infections and increasing ↑ with disease severity) and greater presence of *Adenoviridae*, *Polyomaviridae* and *Propionibacterium* phages. Intestinal tract infections are typically linked to an increase in *Adenoviridae*, *Astroviridae*, *Caliciviridae*, and *Picornaviridae*, and a decrease in *Microviridae* and *Caudovirales*; notably, *Caudovirales* are an exception, showing enrichment only during *Clostridioides difficile* (CD) infection. In the urogenital tract, infections in this area are linked to an increased abundance of *Papillomaviridae* and *Anelloviridae*. These observations highlight how infections can influence the virome across the three major body sites. Created in BioRender. Rebecca Feletti. (2026) https://BioRender.com/.

The following sections, including Table 1 and Figure 2, summarize the most recent and available evidence on the virome characterization across key anatomical compartments that are involved in human infections. Particular attention is given to the respiratory, intestinal, and urogenital tracts, which represent major interfaces between the host and the external environment.

### 7.1. The Virome in Respiratory Infections

The respiratory tract, a primary interface with the external environment, harbors a dynamic and diverse viral community [135,136,137,138]. While traditional diagnostics for respiratory infections focus on a single causative agent or a panel of pathogens in syndromic approach testing, a broader virome perspective reveals a complex viral ecology whose composition is intrinsically linked to host immunity and disease outcomes [139]. Studies on the virome in acute and chronic respiratory illnesses, including COVID-19 and other common infections, are shedding light on these intricate viral–host and viral–viral interactions.

The virome provides a crucial context for understanding the pathogenesis of acute respiratory infections (ARIs) [12,140,141,142,143,144,145,146,147]. Across acute respiratory infections of viral or bacterial origin, the respiratory virome reflects interactions among host immunity, microbial communities, and infection severity. Current evidence is mainly associative; therefore, its role should be described as potentially contributory rather than definitively causal [148,149]. Metagenomic studies in ARI consistently identify a high diversity and richness of eukaryotic viral families (*Paramyxoviridae*, e.g., RSV and human metapneumovirus, *Picornaviridae,* e.g., rhinoviruses, *Orthomyxoviridae,* e.g., influenza, *Adenoviridae*, *Anelloviridae*, *Polyomaviridae*, *Parvoviridae*, *Coronaviridae*, *Herpesviridae*, *Astroviridae* and *Papillomaviridae*) showing correlations between viral abundance and disease severity (e.g., pneumoniae) compared to that of asymptomatic or healthy controls [52,62,136,139,148,150,151,152,153,154,155,156,157,158]. A consistent observation across multiple studies is the expansion of specific viral families—notably *Anelloviridae, Adenoviridae* and *Polyomaviridae*—particularly in severe or immunologically compromised settings, suggesting that acute viral infection may disrupt the virome and reduce immune control over commensal or persistent viruses [52,136,150,151,155,159,160].

In particular, anelloviruses appear to follow a biphasic dynamic, with a transient decrease often observed during the acute infection phase, likely reflecting increased immune activation, followed by an increase in abundance in cases of severe disease or immunodeficiency conditions, consistent with their dependence on host immune control [52]

In parallel, alterations in the bacteriophage community have also been observed, indicating a close interplay between viral and bacterial components of the airway microbiome. Changes in bacteriophage populations are thought to reflect and potentially alter bacterial community structure, thereby contributing to airway inflammation and tissue pathology [52,161,162,163]. Supporting this view, a longitudinal study of children with recurrent respiratory tract infections reported an increased abundance of *Propionibacterium*-associated bacteriophages compared to children with single infection episodes over a six-year period, consistent with persistent alterations in microbial communities [52,159].

The coexistence or exclusion of certain viral species may indicate that competitive and synergistic interactions among viruses may influence both infection dynamics and clinical outcomes [144,158,163]. In SARS-CoV-2 infection, the perturbation of the respiratory virome extends beyond the airways [161,164,165,166,167,168], engaging the gut–lung axis and causing systemic virome alterations detectable in the blood [169]. The elevation of circulating anellovirus load consistently correlates with disease severity and immune exhaustion, reinforcing its potential as a universal biomarker of immune competence across diverse clinical contexts [147,148,169,170].

An interesting recent work reported that a specific viral signature precedes hospital-acquired pneumonia onset in intubated critically ill patients, characterized by a decrease in viral beta diversity (which evaluates differences between communities) evolving into a bacteriophage-dominated lung virome enriched in Caudoviricetes. This was followed by an increase in eukaryotic viruses, among which Alphatorquevirus was the most abundant [128].

From a clinical perspective, these converging findings highlight that virome analysis transcends traditional pathogen detection. Instead of identifying a single etiologic agent, it provides a comprehensive snapshot of the respiratory ecosystem, revealing how viral communities respond to and shape host physiology. Incorporating virome profiling into clinical practice could therefore enhance differential diagnosis, enable risk stratification, and support personalized therapeutic strategies, especially in patients with complex or recurrent respiratory diseases.

### 7.2. The Virome in Intestinal Infections

Local infections affecting the intestinal tract are accompanied by profound alterations in the virome, reflecting the close interplay between enteric viruses, bacteriophages, and the bacterial microbiota. Across infections of different etiologies—whether viral, parasitic, or bacterial—a common pattern emerges: acute or chronic intestinal alterations are associated with a shift from a bacteriophage-dominated community characterized by greater viral diversity and compositional stability to a community with an increased representation of eukaryotic viruses and less stability.

In acute viral gastroenteritis, for instance, rotavirus infection [171] induces a dramatic restructuring of the intestinal virome. Comparative analyses of fecal samples from children infected with Rotavirus A and uninfected controls, using alpha diversity (which assesses species richness within a community) and beta diversity (which evaluates differences between communities), revealed distinct beta diversity profiles despite similar alpha diversity values [172]. Infected individuals exhibited a marked predominance of eukaryotic viruses—mainly from the *Adenoviridae, Astroviridae, Caliciviridae*, and *Picornaviridae* families—accompanied by a depletion of bacteriophages belonging to *Microviridae* and *Caudovirales* [172]. This replacement of bacteriophage-rich communities by enteric eukaryotic viruses suggests that active viral replication may drive a shift in viral composition, likely through inflammation-mediated disruption of bacterial hosts and niche competition. Our understanding of the inter-kingdom interactions is currently emerging from various organism classifications and their prospective influence on shaping the gut micro-ecology. For instance, the role of non-bacterial agents, especially viral and fungal agents in the pathogenesis of necrotizing enterocolitis (NEC) or NEC-like symptoms has recently been postulated [173]. Specifically, eukaryotic viruses are observed to cause clinical illness in newborns, in contrast to the other components of the gut virome, namely bacteriophages [173].

Comparable patterns of virome remodeling are observed in non-viral intestinal diseases. Infections by the protozoan *Giardia duodenalis*, for example, are associated with significant changes in the bacteriophage population, particularly those targeting members of the *Proteobacteria* phylum, such as *Escherichia coli*, *Salmonella*, *Shigella*, and *Yersinia* [129]. The selective enrichment of these bacteriophages in Giardia-positive individuals implies that parasitic infection modifies the gut environment in a way that favors specific viral–bacterial interactions, with potential effects on microbial community structure and shaping host inflammatory responses.

Similarly, *Clostridioides difficile* infection (CDI)—often associated with antibiotic-induced disruption of the gut bacterial populations [130,174]—shows a distinct signature of virome imbalance. CDI patients display an expansion of *Caudovirales* phages and elevated *Anelloviridae* abundance, coupled with reduced overall diversity and a depletion of *Microviridae* species [130,131,132,175]. The normal negative correlation observed between *Caudovirales*, *Microviridae*, and *Anelloviridae* in healthy individuals is lost during infection, indicating a breakdown in the equilibrium of the intestinal virome [175]. The profound disruption of virome homeostasis observed in CDI underpins the clinical efficacy of fecal transplantation in restoring both bacterial and viral community balance, and supports the growing interest in bacteriophage therapy as a targeted approach to counteract *C. difficile* overgrowth while preserving ecosystem stability [21,175,176].

Taken together, these observations highlight the virome as a sensitive and dynamic component of intestinal homeostasis. Whether triggered by viral, parasitic, or bacterial pathogens, infection-induced disturbances consistently manifest as altered viral community structure, loss of bacteriophage-mediated microbial regulation, and over-representation of specific viral taxa. Such signatures may suggest the virome’s dual role as both a marker and active participant of intestinal ecosystem health, offering new perspectives for the diagnosis and understanding of gut-associated diseases.

### 7.3. The Virome in Urinary and Genital Infections

The long-standing belief that the genito-urinary tract is sterile has been overturned by metagenomic research revealing a diverse microbial ecosystem that includes a resident virome [177]. Virome profiling is now providing crucial insights into the etiology, progression and persistence of urinary and genital tract infections, supporting a paradigm shift in urogenital infectious disease research. Metagenomic studies have revealed that both urinary and genital environments harbor a rich and dynamic viral ecosystem, which includes bacteriophages as well as eukaryotic viruses [133]. Understanding how these viral communities interact with local microbiota and the host immune system is now recognized as essential for characterizing disease onset, progression and persistence.

Across both the urinary tract and the cervicovaginal environment, bacteriophages constitute the major component of the virome and appear to contribute to microbial homeostasis. In the urine, approximately 10^7^ viruses per milliliter have been reported, the majority belonging to bacteriophage taxa [178,179]. Eukaryotic viruses such as non-oncogenic HPVs are also commonly detected, with prevalence reaching up to 95% even in individuals without urinary tract infection (UTI) symptoms [180]. These observations undermine the assumption that viral detection necessarily reflects active pathology and instead suggest persistence and commensalism as common features of the urogenital virome.

Clinical conditions are associated with distinct shifts in viral community structure, particularly within the vaginal microbiome. While bacteriophages dominate in eubiotic states—closely mirroring the presence of protective *Lactobacillus* species—conditions such as vaginitis and bacterial vaginosis exhibit increased proportions of pathogenic eukaryotic viruses, notably *Papillomaviridae*, accompanied by reduced viral alpha diversity and increased beta diversity [133,134]. These viral changes occur alongside alterations in bacterial communities, highlighting a strong functional interdependence between bacteriophage dynamics and the stability of the bacterial microbiota. In contrast, viral shifts during urinary infections tend to be less pronounced than bacterial changes, suggesting greater stability of the urinary virome under inflammation [180].

Beyond their role in microbial balance, viral communities within the cervicovaginal tract have been implicated in the persistence and oncogenic evolution of HPV infections. Anelloviruses, including TTV, show significant correlation with genital HPV-positive status and have been linked to enhanced tumor progression in HPV-associated cancers [181,182]. These viruses are considered markers of immune dysregulation and may facilitate a co-pathogenic environment that promotes cervical cancer progression. Moreover, trans-kingdom interactions involving bacteriophages and their bacterial hosts are emerging as potential contributors to viral oncogenesis and mucosal immunity [183].

Taken together, these findings may indicate that the urogenital virome is not a passive occupant but an active component influencing microbial equilibrium, infection susceptibility and disease outcome [113,133,184]. The shared presence of abundant bacteriophage communities, the widespread yet often non-pathological detection of eukaryotic viruses, and the interplay between viral shifts and local changes represent key commonalities across the urinary and genital compartments [178,179]. Clinically, this calls for a refined interpretation of viral presence—particularly for HPV and TTV—and for the development of virome-based diagnostic markers capable of differentiating transient colonization from pathogenic progression. Furthermore, the virome represents a promising therapeutic target: manipulating bacteriophage populations to restore microbial balance could offer innovative treatment strategies for recurrent UTIs, vaginitis and HPV-associated diseases.

## 8. Conclusions and Future Perspectives

This review highlights the virome as an integral component of host–microbial interactions in infectious diseases. Our exploration across various body districts and infection types reveals several key themes. In chronic infections like HIV, the proliferation of ubiquitous viruses, particularly anelloviruses, serves as a potential indicator of host immune system compromise. This suggests that the virome’s composition can provide a real-time snapshot of the host’s immunological state, offering valuable prognostic information. Moreover, virome analysis highlights the frequent occurrence of co-infections (e.g., rhinovirus with SARS-CoV-2, TTV with HPV) that can influence disease severity and progression. This virome-based perspective shifts our understanding from a single causative agent to a community of viruses whose combined effects determine clinical outcomes.

The bacteriophage community—the largest fraction of the virome—is not a passive bystander. It can actively shape the bacterial microbiome, and its alteration is a hallmark of many diseases. The dramatic shifts in bacteriophage populations observed in CDI and *M. pneumoniae* infections underscore the critical role of these viral–bacterial interactions in disease pathogenesis.

A major limitation of current virome research is the predominance of associative data without adequate mechanistic integration. While many studies report correlations between virome composition and clinical outcomes, the underlying immune-mediated mechanisms driving these associations remain poorly understood. This highlights the need for integrating virome profiling with immune correlates to better elucidate causal pathways of virome modulation in infectious diseases.

The field of virome research is still in its nascent stages, and significant challenges remain. The vast majority of viral sequences remain unclassified, representing a “dark matter” that requires more comprehensive characterization. However, the insights gained thus far point to several promising directions for future research and clinical applications. The development of virome-based biomarkers could improve diagnostics. A viral signature, such as an anelloviruses’ load, can be a standard clinical metric for assessing immune status or predicting disease severity. Understanding the virome opens the door to novel therapeutic strategies. For instance, bacteriophage therapy is showing great potential in selectively modulating the bacterial microbiome in conditions like CDI, or to combat antibiotic-resistant infections.

While the virome’s role is an active area of investigation in chronic inflammatory and autoimmune conditions, metabolic disorders and cancer progression, this review focused on infectious diseases, and future research is needed to explore these connections and elucidate the virome’s systemic influence on human physiology.

An important and still underexplored area concerns chronic bacterial and parasitic infections. Infections such as tuberculosis, *H. pylori* gastritis, leishmaniosis and chronic helminthic infestations involve long-term host–pathogen coexistence, immune tolerance and incomplete microbial clearance, processes that are likely influenced by baseline microbial ecosystem stability. In this context, virome composition may provide additional insights into infection control, progression to tissue damage, or post-treatment relapse.

Future investigations could benefit from longitudinal and compartment-specific approaches integrating virome profiling with the bacterial microbiome and host immune parameters to better define its clinical relevance. In this context, virome profiling may also support longitudinal monitoring in settings such as chronic infection, intensive care, and transplantation, where shifts in viral communities could reflect treatment response or changes in immune control, corroborating its potential as a complementary clinical biomarker.

However, the translation of virome profiling into routine clinical practice remains challenging, particularly in low- and middle-income countries. Key barriers include the high costs of metagenomic sequencing, the need for specialized infrastructure and technical expertise, and the complexity of data analysis and interpretation. Addressing these limitations will require the development of cost-effective, standardized, and scalable approaches to ensure broader accessibility and clinical applicability, especially in settings where infectious diseases are most prevalent.

Continued improvements in sequencing technologies, bioinformatics pipelines and standardized protocols will be essential to fully catalog the virome and unlock its full potential for clinical and scientific discovery, highlighting the virome’s utility as both a diagnostic and epidemiological resource.

## Figures and Tables

**Table 1:** Main changes in human virome reported across infectious diseases.

Infection	Main Virome Change Observed	Proposed Implication	References
Acute respiratory infections/hospital-acquired pneumonia	Reduced viral diversity evolving toward bacteriophage-dominated community followed by eukaryotic virus expansion	Virome may reflect inflammatory respiratory tract ecosystem and could be associated with prognosis rather than pathogen identity	[52,128]
*G. duodenalis* infection	Enrichment of bacteriophages targeting Proteobacteria	Parasite-driven remodeling of the bacterial microbiome	[129]
*C. difficile* infection	Major shifts in bacteriophage populations	Bacteriophage–bacteria interactions can contribute to persistence and recurrence	[130,131,132]
Chronic viral infections (HIV, HBV, HCV, HPV)	Expansion of *Anelloviridae*	Potential marker of impaired immune surveillance rather than direct pathogenicity	[59,111,112]
Urogenital infection	Shift from bacteriophage dominance to pathogenic eukaryotic viruses (e.g., *Papillomaviridae*) in vaginitis and bacterial vaginosis	Virome indicates ecosystem alteration but not etiologic diagnosis; potential for bacteriophage-based therapy	[133,134]

Footnote: HIV, Human immunodeficiency virus; HBV, Hepatitis B virus; HCV, Hepatitis C virus; HPV, Human papillomavirus.

## Data Availability

No new data were created or analyzed in this study. Data sharing is not applicable to this article.

## Supplementary Materials

The following supporting information can be downloaded at: https://www.mdpi.com/article/10.3390/microorganisms14050969/s1, Table S1. Key concepts of the microbiome.

## Abbreviations

HIV	Human immunodeficiency virus
HBV	Hepatitis B virus
HCV	Hepatitis C virus
HPV	Human papillomavirus
CSF	Cerebrospinal fluid
EBV	Epstein–Barr virus
CMV	Cytomegalovirus
TTV	Torque teno virus
HERV	Human endogenous retroviruses
BKPyV	BK polyomavirus
MCV	Molluscum contagiosum virus
ARIs	Acute respiratory infections
NEC	Necrotizing enterocolitis
CDI	Clostridioides difficile infection
UTI	Urinary tract infection
ITS	Internal transcribed spacer
