# Supplementary material for: The Human Virome in Infectious Diseases: Insights from Chronic and Acute Infections Across Body Sites—A Narrative Review
Source: Microorganisms. 2026 Apr 25;14(5):969. doi: 10.3390/microorganisms14050969 (PMC13209241; doi:10.3390/microorganisms14050969)
Supplement: Supplementary file 1 [file microorganisms-14-00969-s001.zip › Table S1.pdf]

Table S1. Key concepts of the microbiome.

Concept	Simplified Definition
Microbiota	The community of microorganisms (bacteria, viruses, fungi, archaea, parasites) living in a body site
Microbiome	The total genetic material of all microorganisms present (DNA/RNA)
Bacteriome	Bacterial component of the microbiome
Archaeome	Archaea present in humans
Virome	All viruses present (eukaryotic, bacteriophages, archaeal viruses)
Phageome	Subset of the virome composed of bacteriophages
Mycobiome	Fungal community (yeasts and filamentous fungi)
Commensalism	Microorganisms present without causing apparent harm
Persistent viruses	Viruses that remain long-term in the host (e.g., herpesviruses, anelloviruses)
Alpha diversity	Measure of differences between microbial communities within a single sample
Beta diversity	Measure of differences between microbial communities across samples
Viral abundance	Relative quantity of specific viruses or viral families
